# The shadow of a doubt? Evidence for perceptuo-motor linkage during auditory and audiovisual close-shadowing

**DOI:** 10.3389/fpsyg.2014.00568

**Published:** 2014-06-24

**Authors:** Lucie Scarbel, Denis Beautemps, Jean-Luc Schwartz, Marc Sato

**Affiliations:** CNRS, Grenoble Images Parole Signal Automatique-Lab, Speech and Cognition Department, UMR 5216, Grenoble UniversityGrenoble, France

**Keywords:** speech perception, speech production, audiovisual speech perception, close-shadowing, sensorimotor interactions

## Abstract

One classical argument in favor of a functional role of the motor system in speech perception comes from the close-shadowing task in which a subject has to identify and to repeat as quickly as possible an auditory speech stimulus. The fact that close-shadowing can occur very rapidly and much faster than manual identification of the speech target is taken to suggest that perceptually induced speech representations are already shaped in a motor-compatible format. Another argument is provided by audiovisual interactions often interpreted as referring to a multisensory-motor framework. In this study, we attempted to combine these two paradigms by testing whether the visual modality could speed motor response in a close-shadowing task. To this aim, both oral and manual responses were evaluated during the perception of auditory and audiovisual speech stimuli, clear or embedded in white noise. Overall, oral responses were faster than manual ones, but it also appeared that they were less accurate in noise, which suggests that motor representations evoked by the speech input could be rough at a first processing stage. In the presence of acoustic noise, the audiovisual modality led to both faster and more accurate responses than the auditory modality. No interaction was however, observed between modality and response. Altogether, these results are interpreted within a two-stage sensory-motor framework, in which the auditory and visual streams are integrated together and with internally generated motor representations before a final decision may be available.

## INTRODUCTION

An old and classical debate in speech communication concerns the possible motor implication in speech perception and, more generally, the auditory vs. motor nature of the speech code. The heart of the debate relies in the existence and possible functional link between auditory and motor representations in both speech perception and speech production. Auditory theories of speech perception, such as the “Acoustic Invariance Theory” from Stevens and Blumstein (1978) or the “Adaptative Variability Theory” from Lindblom and Maddieson (1988) and Lindblom (1990) assume that speech perceptual processing and categorization are based on acoustic cues and auditory representations, with no need to call for any knowledge about the way the articulatory system produces the sound (Diehl et al., 2004). Conversely, the motor theory of speech perception (Liberman and Mattingly, 1985) and its direct realist variant (Fowler, 1986) claim that there is a crucial role of the motor system in speech perception, and consider that speech perception involves recovery of the stimulus cause, either physically (recovering the configuration of the vocal tract, in Fowler’s direct realist theory) or biologically/cognitively (inferring motor commands in Liberman and Mattingly, 1985). More recently, a number of perceptuo-motor theories attempted various kinds of syntheses of arguments by tenants of both auditory and motor theories, proposing that implicit motor knowledge and motor representations are used in relationship with auditory representations and processes to elaborate phonetic decisions (Skipper et al., 2007; Schwartz et al., 2012).

It is worth noting that the question of whether articulatory processes mediate speech perception under normal listening conditions still remains vigorously debated (e.g., Hickok and Poeppel, 2007; Lotto et al., 2009; Scott et al., 2009; D’Ausilio et al., 2012; Schwartz et al., 2012). On the one hand, damage to motor speech areas in Broca’s aphasic patients does not produce clear deficits in speech perception (e.g., Hickok et al., 2011) and studies using transcranial magnetic stimulation (TMS) also challenge a possible mediating role of the motor system in speech processing under normal listening conditions (Sato et al., 2009; D’Ausilio et al., 2011). On the other hand, an increasing number of neuroanatomical and neurophysiological studies suggest that there is indeed an active relationship between auditory and motor areas, both in speech perception and speech production. Indeed, brain imaging studies [functional magnetic resonance imaging (fMRI) or magnetoencephalography (MEG)] repeatedly showed the involvement of areas typically engaged in the speech production process (the left inferior frontal gyrus, ventral premotor cortex, primary motor cortex, somatosensory cortex) during various speech perception tasks (e.g., Binder et al., 2004; Möttönen et al., 2004; Wilson and Iacoboni, 2006; Grabski et al., 2013a), particularly in adverse conditions (e.g., noise: Zekveld et al., 2006; or foreign accent: Callan et al., 2004). TMS experiments confirmed the involvement of the motor system in speech perception, both auditory and audiovisual (Fadiga et al., 2002; Wilson et al., 2004). However, evidence for a perceptuo-motor link in the human brain is not a proof that this link plays a functional role for processing speech inputs. Some neurophysiological evidence based on the use of TMS provided some evidence that perturbations of the motor system could lead to slight but significant modifications of the speech perceptual decision process (e.g., Meister et al., 2007; D’Ausilio et al., 2009; Möttönen and Watkins, 2009, 2012; Sato et al., 2009; Grabski et al., 2013b), but the perturbations are small and sometimes difficult to interpret.

In an influential review about the motor theory of speech perception, Galantucci et al. (2006) summarize different arguments to argue that “perceiving speech is perceiving gestures.” One first argument comes from co-articulation effects and the fact that the acoustic properties of speech sounds are not invariant but context dependent. Since the correspondence between sounds and phonemes can be far from transparent, this led researchers to propose intended gestures as less invariant and as the ultimate objects of speech perception (see Liberman and Mattingly, 1985). Other arguments derive from close-shadowing effects and multisensory speech perception. Let us focus on these last two arguments, which will provide the basis for the present study.

Close-shadowing, which is an experimental technique in which subjects have to repeat speech immediately after hearing it, provides a natural paradigm for displaying perceptuo-motor links. In their pioneer study, Porter and Castellanos (1980) compared reaction times (RTs) in two speech perception tasks involving vowel-consonant-vowel (VCV) syllables (/aba/, /apa/, /ama/, /aka/, /aga/): in the first task, participants had to shadow the VCV they heard, that is to reproduce it orally as quickly as possible. They first produced the initial vowel and then shifted to the consonant as soon as they could perceive and identify it. The second task was a simple choice task: subjects had to shadow the initial vowel and, when stimulus changed into any consonant, they had to shift to /ba/ whatever the consonant, as quickly as possible. The authors found that RTs were of course faster in the simple task than in the shadowing task involving decision, but this difference was not very large (between 30 and 60 ms). Galantucci et al. (2006) compared those results with RTs found in Luce (1986), who used the same kind of paradigms (simple choice vs. multiple choice task, with comparable stimuli), but in responding by pressing a key rather than orally producing a response (in the choice task, participants had to press the key corresponding to the syllable they heard, and in the simple choice task they had to press a given key, whatever they heard). In Luce (1986) differences between RTs in the two tasks were larger than those in Porter and Castellanos’s (1980) close-shadowing tasks (100/150 vs. 30/60 ms). This difference was interpreted by Galantucci et al. (2006) the assumption that, since perceiving speech is perceiving gestures, gesture perception will directly control speech response and make it faster. Later on, Fowler et al. (2003) published a study based on Porter and Castellanos’s (1980) work, in which the participants had to shadow syllables in a “one choice task” and in a “multiple choice task” with three types of stimuli: /apa/, /aka/, and /ata/. In the “one choice task,” participants were assigned to one of the three VCVs, shadowing the initial /a/ and instructed to switch toward their own consonant as soon as the stimulus consonant was presented, but independently of the identity of the stimulus consonant. In the “multiple choice task,” participants simply had to shadow all VCVs. As in Porter and Castellanos (1980), they found that participants had shorter RTs in the simple choice task than in the multiple choice task. In the simple choice task, they also compared RTs between the three groups of subjects (one per assigned syllable) and they found that participants had shorter RTs when presented stimuli matched with their own syllable. These results are interpreted by Fowler et al. (2003), as well as by Galantucci et al., 2006, as suggesting that acoustic stimuli perceived as articulatory gestures would provide a prior “response goal” therefore modulating response times depending on the compatibility between stimulus and requested response.

Concerning multisensoriality on speech perception, it is known since long that lip-reading is helpful for understanding speech. Apart from the importance of lip-reading for hearing impaired subjects, normal-hearing subjects are able to lip-read (Cotton, 1935) and we know at least since Sumby and Pollack (1954) that the visual modality enhances auditory speech comprehension in noise. Shadowing experiments have actually also been exploited to assess audiovisual interactions in speech perception, though with no temporal constraint. Indeed, Reisberg et al. (1987) studied the audiovisual benefits in shadowing foreign language stimuli or linguistically complex utterances. In two experiments, he tested two groups of English participants to measure accuracy in production; participants were supposed to shadow French or German sentences, in audio vs. audiovisual conditions. Participants obtained significantly better scores – in terms of global accuracy of repetition – in the audiovisual condition compared with the audio condition. Then he tested one group of English participants who had to shadow English stimuli spoken with a Belgian accent, in audio and audiovisual conditions, in three experiments: one with simple phrases, one with more complex phrases and one with rare words. Once again, participants had better scores in the audiovisual condition. Then, Davis and Kim (2001) tested accuracy scores in repetitions of Korean phrases, by naïve English speakers, in a delayed shadowing experiment. Participants had to repeat stimuli at the end of the signal, in an audio and an audiovisual condition. After the repetition task, participants listened to a number of stimuli and had to decide whether they had already heard the stimuli or not. In both tasks, accuracy was better in the audiovisual condition.

However, all the audiovisual shadowing experiments do not deal with close-shadowing, hence they lack information about the dynamics of the decision process in relation with perceptuo-motor relationships. On the other side, close-shadowing experiments never involve audiovisual inputs, hence they lack information about the relationship between audiovisual interaction processes and perceptuo-motor interaction processes in phonetic categorization. Therefore audiovisual close-shadowing is the purpose of the present study in order to test audiovisual and perceptuo-motor interactions in an integrated paradigm.

One experiment was performed by two groups of French participants and focused on a comparative assessment of the accuracy and speed of oral vs. manual responses to auditory vs. audiovisual speech stimuli (VCV syllables). The speech stimuli were presented without acoustic noise for the first group (Group A in the remainder of this paper) or with acoustic noise in the second one (Group B in the remainder of this paper). Our hypotheses were that (1) oral responses should be faster than manual responses, in agreement with previous studies on close-shadowing reported here above, and that (2) responses to audiovisual stimuli should be faster and more accurate than those to audio-only stimuli, at least in noise. An additional question concerns the possibility of interaction between these two components, evaluating whether the effect of vision is different from one modality of response (oral) to the other (manual). The responses to these questions will then be discussed in relationship with the debates about multisensory and perceptuo-motor interactions in speech perception.

## MATERIALS AND METHODS

### PARTICIPANTS

Two groups of respectively 15 and 14 healthy adults, native French speakers, participated in the experiment (Group A: 10 females; mean age: 29 years, age range: 20–39 years – Group B: 11 females; mean age: 24 years, age range: 19–34 years). All participants had normal or corrected-to-normal vision and reported no history of speaking, hearing or motor disorders. The experiment was performed in accordance with the ethical standards laid down in the 1964 Declaration of Helsinki.

### STIMULI

Multiple utterances of /apa/, /ata/, and /aka/ VCV syllables were individually produced by a male native French speaker (who did not participate in the experiment) in a sound-attenuated room. These three syllables were selected according to the distinct place of articulation of the consonant (stop bilabial /p/, alveolar /t/, and velar /k/) and to ensure a gradient of visual recognition between these syllables (with notably the bilabial /p/ consonant known to be more visually salient than alveolar /t/ and velar /k/ consonants). The syllables were audiovisually recorded using an AKG 1000S microphone and a high-quality digital video camera placed in front of the speaker zooming his face.

The corpus was recorded with the objective to obtain four different occurrences of /apa/, /ata/, and /aka/ with various durations of the initial /a/ vowel (i.e., 0.5s, 1s, 1.5s, and 2s). This was done in order to present participants with stimuli in which the onset of the consonant to categorize would occur at an unpredictable temporal position. To this aim, the speaker was asked to maintain the production of the initial vowel while expecting a visual “go” signal. The speaker produced 48 stimuli (4 initial durations × 3 types of syllables × 4 repetitions). One utterance was selected for each stimulus type and each initial vowel duration so as to obtain 12 stimuli. Then, to remove potential irrelevant acoustic differences between the stimuli, the occurrences of /apa/, /ata/, and /aka/ for a given expected initial duration were cut at their onset to equalize duration of the first vowel. Similarly, duration of the final vowel was equalized at 240 ms for all the 12 stimuli.

The audio tracks of the stimuli were sampled at 44.1 kHz and presented without noise in Group A. In Group B, the 12 stimuli were mixed with white noise, low pass filtered at -6 dB/oct, with a signal to noise ratio at -3 dB (the signal energy being defined from burst onset to the end of the vowel). In the audiovisual modality of the experiment, the video stream consisted in 572-by-520 pixel/images presented at a 50 Hz rate with the speaker’s full face presented with blue lips to enhance lips movement perception.

### EXPERIMENTAL PROCEDURE

The experiment consisted of two categorization tasks: close-shadowing in one case, where the responses were provided orally, by repeating as quickly as possible the presented speech syllables; manual decision in the other case, where the responses were provided manually, by pressing as quickly as possible the appropriate key. The stimuli to categorize consisted in /apa/, /ata/, and /aka/ syllables.

**FIGURE 1:**
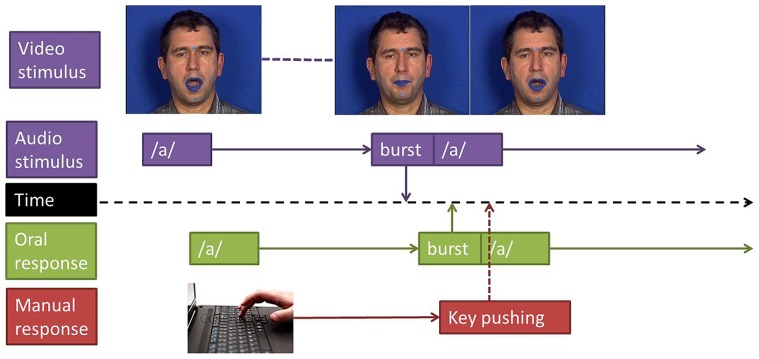
**Experimental design.** Reaction times where measured between stimulus’ and responses’ burst (plosion release) for oral responses and between stimulus’ burst and key pushing for manual responses.

Participants were told that they would be presented with /apa/, /ata/, or /aka/ syllables, displayed either auditorily or audiovisually. In the close-shadowing task they were instructed to categorize and repeat each syllable as quickly as possible. To do so, they were asked to shadow the initial /a/ vowel and, when the stimulus changed to consonant, to immediately categorize and repeat the perceived CV syllable (/pa/, /ta/, or /ka/; see **Figure 1**). In the manual decision task, participants were instructed to categorize each syllable by pressing as quickly as possible with their dominant hand one of three keys respectively corresponding to /apa/, /ata/, or /aka/. The order of keys was counterbalanced across participants.

For each task (oral vs. manual response) and each modality (auditory vs. audiovisual), 16 occurrences of /apa/, /ata/, and /aka/syllables were presented in a fully randomized sequence of 48 trials. The order of task and modality of presentation was fully counterbalanced across participants.

Both groups performed the experiment in a soundproof room. Participants sat in front of a computer monitor at a distance of approximately 50 cm. The acoustic stimuli were presented at a comfortable sound level, with the same sound level set for all participants. While in Group A, the presentation of acoustic stimuli was done with a loudspeaker, the presentation of acoustic stimuli was done with earphones in Group B. This was required because of noisy stimuli, making acoustic processing complex and inaccurate if stimulus and response were mixed. The Presentation software (Neurobehavioral Systems, Albany, CA, USA) was used to control the stimulus presentation and to record key responses in the manual task. All participants’ productions were recorded using an AKG 1000S microphone for off-line analyses, with a system ensuring synchrony between the stimulus presented to the participant and the participant’s response. A brief training session preceded each task. The total duration of the experiment was around 30 min.

### ACOUSTIC ANALYSES

In order to calculate RTs and the percentage of correct responses in the speech shadowing task, acoustic analyses of participants’ productions were performed using Praat software (Boersma and Weenink, 2013). A semi-automatic procedure was first devised for segmenting participants’ recorded productions. Based on minimal duration and low intensity energy parameters, the procedure involved the automatic segmentation of each utterance based on an intensity and duration algorithm detection. Then, for each presented stimulus, whatever the modality of presentation and response, an experimenter coded the participant’s response and assessed whether it was correct or not.

Reaction times were estimated in reference to the burst onset of the stop consonant to categorize. In the manual decision task, the response instant was provided by the Presentation software, giving the instant when the key was pressed. In the close-shadowing tasks, the response time was provided by the burst onset of the stop consonant uttered by the participant in response to the stimulus, burst detection being realized by looking at the subject’s production and inspecting waveform and spectrogram information with the Praat software. RTs were computed only for correct responses: omissions or any types of errors (replacing a consonant by another or producing two consonants or two syllables in the close-shadowing task) were excluded. The timelines of stimuli and responses, including description of the way response times were measured in both tasks, are displayed in **Figure 1**.

### DATA ANALYSES

For each group, the percentage of correct responses and median RTs were individually determined for each participant, each task, each modality, and each syllable. Two repeated-measure ANOVAs were performed on these measures with the group (Group A with clear stimuli vs. Group B with noisy stimuli) as a between-subject variable and the task (close-shadowing vs. manual decision), the modality (auditory vs. audiovisual AV) and the syllable (/apa/ vs. /ata/ vs. /aka/) as within-subjects variables.

## RESULTS

For all the following analyses, the significance level was set at *p* = 0.05 and Greenhouse–Geisser corrected (in case of violation of the sphericity assumption) when appropriate. All reported comparisons refer to *post hoc* analyses conducted with Bonferroni tests.

### REACTION TIMES

**FIGURE 2:**
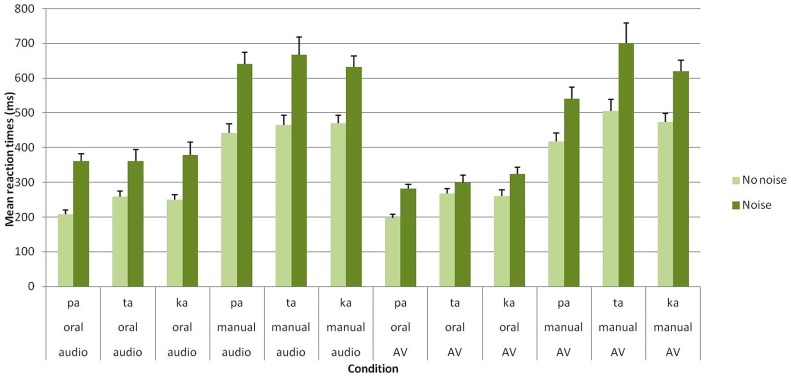
**Mean RTs (in ms) of correct identification for /apa/, /ata/, and /aka/ syllables in each group (Group A without noise vs. Group B with noise), task (oral vs. manual response) and modality of presentation (auditory vs. audiovisual).** Error bars represent standard errors of the mean.

As expected, the main effect of group was significant [*F*(1,27) = 24.38; *p* < 0.001], with faster RTs observed for clear stimuli in Group A compared to noisy/ambiguous stimuli in Group B (351 vs. 484 ms). Crucially, the main effects of task [*F*(1,27) = 151.70; *p* < 0.001] and modality [*F*(1,27) = 14.79; *p* < 0.001] were also found to be reliable. For the task, oral responses were faster than manual responses (286 vs. 545 ms). Regarding the modality, responses were faster in the audiovisual compared to the auditory modality (405 vs. 425 ms). Importantly, a significant group × modality [*F*(1,27) = 21.74; *p* < 0.001] further show that the beneficial effect of audiovisual presentation occurred with noisy stimuli in Group B (461 vs. 507 ms) but not with clear stimuli in Group A (354 vs. 349 ms; see **Figure 2** and **Table 1**).

**Table 1:** Significant effects and interactions for all variables.

	Reaction times	Percentage of correct responses
Group effect	*p* < 0.001	*p* < 0.001
Task effect	*p* < 0.001	*p* < 0.001
Modality effect	*p* < 0.001	*p* < 0.001
Group × Modality	*p* < 0.001	*p* < 0.001
Goup × Task	*p* < 0.001	*p* < 0.001
Syllable effect	*p* < 0.001	*p* < 0.001
Syllable × Modality	*p* < 0.001	n.s.
Syllable × Group	n.s.	*p* < 0.001
Syllable × Task	n.s.	*p* < 0.001
Group × Task × Syllable	n.s.	*p* < 0.001
Modality × Task × Syllable	*p* < 0.005	n.s.

In sum, the above-mentioned results thus replicate and extend previous studies on speech shadowing (references) by demonstrating a clear advantage of oral responses with both clear and noisy stimuli. In addition, compared to unimodal auditory stimuli, audiovisual stimuli led to faster RTs but only with noisy stimuli. Interestingly, no interaction was found between these two effects thus suggesting they occurred independently.

It should be however, mentioned that these effects also appear dependent on the perceived speech syllable. Overall, significant differences were found between syllables [*F*(2,54) = 9.66; *p* < 0.001], with faster RTs for /apa/ (383 ms) than for /ata/ (438 ms) and /aka/ (424 ms). In addition, a significant syllable × modality interaction was observed [*F*(2,54) = 10.88; *p* < 0.001]. *Post hoc* analyses showed that RTs for /apa/ were faster in the audiovisual compared to the auditory conditions (357 vs. 410 ms), while RTs for /ata/ and /aka/ did not differ in the two modalities (/ata/: 441 vs. 435 ms; /aka/: 418 vs. 430 ms). Finally, a task × modality × syllable interaction was found [*F*(2,54) = 6.49; *p* < 0.005].In the auditory modality, no significant RT differences were observed between syllables for both oral (/apa/: 282 ms; /ata/: 308 ms; /aka/: 312 ms) and manual responses (/apa/: 538 ms; /ata/: 563 ms; /aka/: 549 ms). However, in the audiovisual modality, faster oral RTs occurred for /apa/ compared to /ata/ and /aka/ (/apa/: 237 ms; /ata/: 283 ms; /aka/: 291 ms) while faster manual RTs occurred for /apa/ compared to /aka/ and for /aka/ compared to /ata/ (/apa/: 476 ms; /ata/: 599 ms; /aka/: 544 ms). Taken together, these results likely indicate that visual information processing depends on the level of visual specificity of the presented consonant, with notably a clear advantage for /apa/ syllable (including a bilabial stop consonant). No other effect or interaction were found to be significant.

### PERCENTAGE OF CORRECT RESPONSES

**FIGURE 3:**
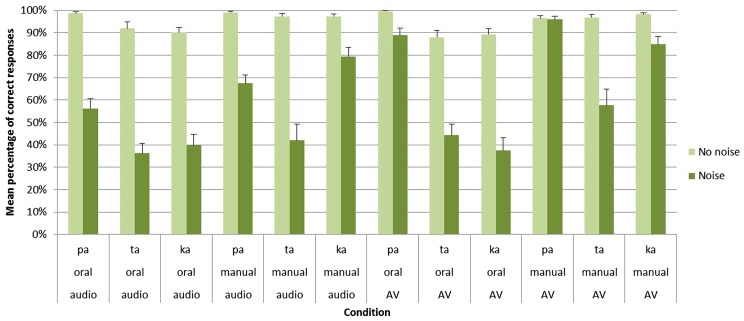
**Mean percentage of correct identification for /apa/, /ata/, and /aka/ syllables in each group (Group A without noise vs. Group B with noise), task (oral vs. manual response) and modality of presentation (auditory vs. audiovisual).** Error bars represent standard errors of the mean.

The main effect of group was significant [*F*(1,27) = 266.28; *p* < 0.001] with a higher percentage of correct responses for clear stimuli in Group A (95%) than for noisy stimuli in Group B (61%). Importantly, significant main effects were also found for both the task [*F*1(1,27) = 69.40; *p* < 0.001] and the modality [*F*(1,27) = 52.39; *p* < 0.001]. Concerning the task, an important decrease of correct responses was observed for oral compared to manual responses (73 vs. 85%). As indicated by a significant group × task interaction [*F*(1,27) = 38.67; *p* < 0.001], this effect only appeared with noisy stimuli in Group B (71 vs. 50%) while no differences were observed between oral and manual responses with clear stimuli in Group A (93 vs. 98%). For the modality, the audiovisual modality led to higher correct responses than the auditory modality (82 vs. 75%). Importantly, as indicated by a significant group × modality interaction [*F*(1,27) = 72.36; *p* < 0.001], no differences were observed between the two modalities with clear stimuli in Group A, (96 vs. 95%) whereas with noisy stimuli in Group B the audiovisual modality led to higher correct responses (68%) compared to the auditory modality (53%; see **Figure 3** and **Table 1**).

In sum, for noisy stimuli, these results demonstrate a beneficial effect of audiovisual presentation together with a dramatic increase of errors for oral responses. As for RTs, no interaction was however, found between these two effects.

Apart from these results, several other effect and interactions occurred depending on the perceived syllable. First, the main effect of syllable was reliable [*F*(2,54) = 25.72; *p* < 0.001], with a higher recognition of /apa/ (80%) compared to /aka/ (78%) as well as for /aka/ compared to /ata/ (70%). Second, a group × syllable interaction [*F*(2,54) = 14.43; *p* < 0.001] was found. With clear stimuli in Group A, no differences were observed between the three syllables (/apa/: 98% /ata/: 94% /aka/: 94%) while, with noisy stimuli in Group B, /apa/ (77%) was better recognized than /aka/ (60%) which was itself better recognized than /ata/ (45%). Third, both a task × syllable [*F*(2,54) = 22.30; *p* < 0.001] and a group × task × syllable [*F*(2,54) = 11.98; *p* < 0.001] interactions were observed. With clear stimuli in Group A, no differences were found between the three syllables for both oral (/apa/: 99%; /ata/: 90%; /aka/: 90%) and manual (/apa/: 98%; /ata/: 97%; /aka/: 98%) responses. With noisy stimuli in Group B, /apa/ was better recognized than /ata/ and /aka/ in the oral response mode (/apa/: 73%; /ata/: 40%; /aka/: 39%) while, for manual responses, /apa/ and /aka/ were better recognized than /ata/ (/apa/: 82%; /ata/: 50%; /aka/: 82%). While the three syllables were almost perfectly recognized without noise in Group A, these results demonstrate that for noisy stimuli /pa/ was here the most auditory and visual salient syllable. No other effect, alone or in interaction, were found to be significant.

### CORRELATION BETWEEN REACTION TIMES AND PERCENTAGE OF CORRECT RESPONSES

**FIGURE 4:**
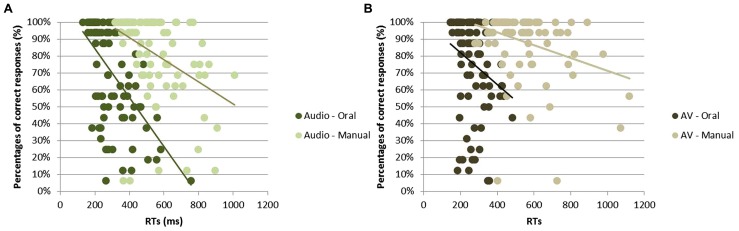
**Correlation between RTs (in ms) and correct identification (in %) for all syllables in response to audio stimuli (A) and to audiovisual stimuli (B)**.

For each of the four condition (i.e., oral or manual responses with audio or AV stimuli), a Pearson’s correlation analysis was performed in order to measure the relationship between RTs and percentage of correct responses (with one correlation point computed for each participant and each syllable, irrespective of the group; see **Figure 4**). For all conditions, the higher was the recognition score, the faster was the response; with a negative correlation between RT and response accuracy observed for oral [*r* = -0.56, *t*(85) = 17.20; *p* < 0.001] and manual [*r* = -0.41, *t*(85) = 14.32; *p* < 0.001] responses to audio stimuli as well as for oral [*r* = -0.24, *t*(85) = 14.36; *p* < 0.001] and manual [*r* = -0.32, *t*(85) = 9.83; *p* < 0.001] responses to AV stimuli.

## DISCUSSION

We will focus the Discussion on the effects associated with the two major components of our study: the mode of responses, oral vs. manual, and the modality of presentation, auditory vs. audiovisual, and the way they impacted participants’ responses.

### EFFECT OF TASK: ORAL vs. MANUAL MODE OF RESPONSE

Without noise (Group A), RTs were significantly faster for oral than for manual response (240 vs. 462 ms), with a non-significant decrease in accuracy in the oral response task (93 vs. 98%). RTs in the oral mode are consistent with those found by Fowler et al. (2003; 248 ms) and Porter and Castellanos (1980; 223 ms) in their multiple choice task. Accuracy in the oral mode happens however, to be higher in our study than in Fowler et al. (2003; 86%) and in Porter and Castellanos (1980; 77%) studies. These differences could be due either to the clarity of the provided stimuli or to the sound level at which the presentation was done (the shadowing of the initial vowel leads to a concurrent sound produced by the participant which may hide to a certain extent the perception of the target plosive to identify). The interpretation by both Porter and Castellanos (1980) and Fowler et al. (2003) of the quick response in the oral mode is done in reference to motor theories of speech perception, in which the speech input would be transformed into a motor representation (Liberman and Mattingly, 1985) or would directly be perceived as an orofacial gesture (Fowler and Smith, 1986). This would enable the orofacial system to respond in a highly rapid way, since the percept would already be in the adequate motor format; and more quickly than the manual system which would need a translation stage between decision and response. More generally, these results appear in line with stimulus–response compatibility effects that suggest a common coding in perception and action (for reviews, see Prinz, 1997; Hommel et al., 2001).

However, the observed results with noisy stimuli (Group B) shed a quite new light on this reasoning. Indeed, while RTs stay much faster in close-shadowing (334 vs. 633 ms), accuracy happens to abruptly decrease from the oral to the manual task (50 vs. 71%). This requires modifying the above-mentioned interpretation by Fowler et al. (2003) and Porter and Castellanos (1980) to a certain extent. We will here propose a tentative explanation in the framework of the perceptuo-motor feed forward-feedback model of speech perception proposed by Skipper et al. (2007).

**FIGURE 5:**
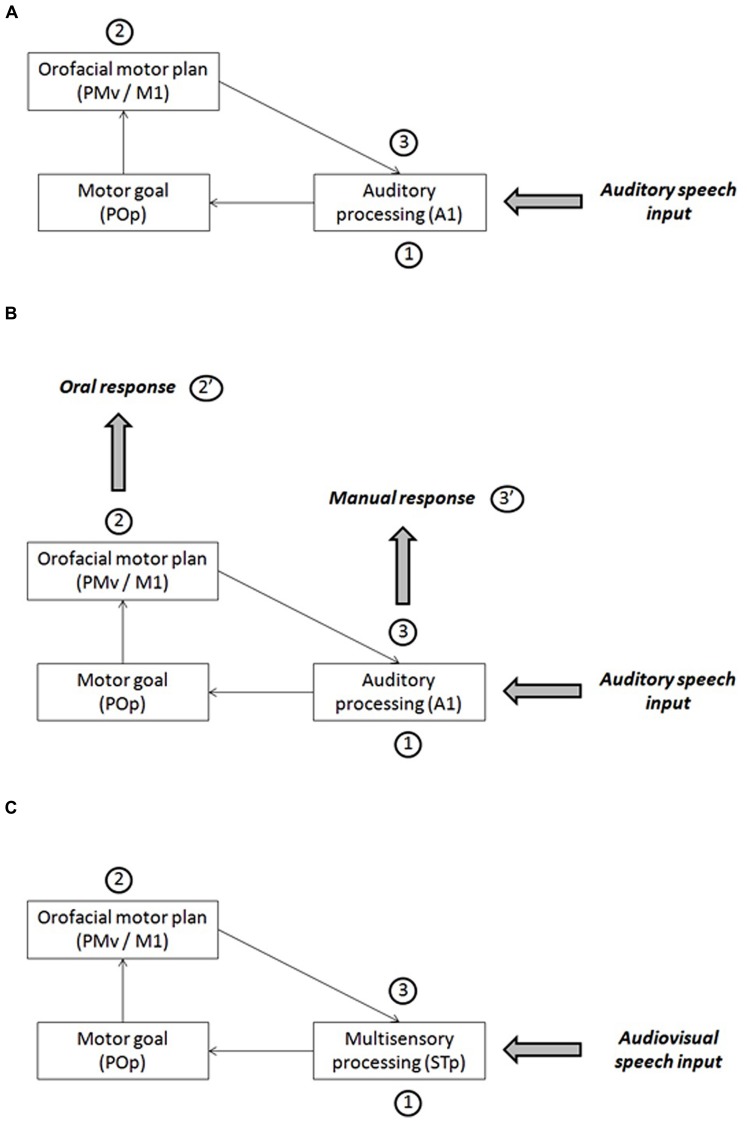
**(A)** A sketch of Skipper et al.’s (2007) model of speech perception for auditory inputs. **(B)** A possible interpretation of the results about the response mode in the framework of Skipper et al.’s (2007) model. **(C)** A sketch of Skipper et al.’s (2007) model of speech perception for audiovisual inputs.

Skipper et al. (2007) propose a speech perception model that they refer to the “analysis-by-synthesis” approach (Halle and Stevens, 1959; Stevens and Halle, 1967; see a review in Bever and Poeppel, 2010). This model involves a processing loop between auditory and motor areas in the human brain (**Figure 3A**). After an initial stage of auditory processing (primary auditory cortex, A1, and further processing in the secondary cortex and associative areas: stage 1 in **Figure 5A**), the auditory cortex would generate a phonemic hypothesis associated with articulatory goals (in the pars opercularis of the inferior frontal gyrus, POp). Then motor commands corresponding to this initial prediction would be stimulated (in the ventral premotor cortex, PMv, and primary motor cortex, M1: stage 2 in **Figure 5A**), leading to the production of an efferent copy sent back to the auditory cortex in order to be compared with the auditory input (stage 3 in **Figure 5A**).

This model could be used as a basis for attempting to interpret our own data (**Figure 5B**). For this aim, we assume that oral and manual responses are generated at two different stages in the processing loop. Oral responses would be generated at stage 2, in line which the assumption by Porter and Castellanos (1980) or Fowler et al. (2003). When the information from the auditory cortex would have been transferred to the POp and generate motor commands in the motor cortex (feedforward strand), the orofacial system, already pre-activated since the beginning of the close-shadowing experiment to allow the participant to answer as quickly as possible, would generate an oral answer produced by these motor commands (stage 2’ in **Figure 5B**). This makes the oral answer faster, but it also happens to be inaccurate, which is in line with the proposal by Skipper et al. (2007) that it is only a first hypothesis (possibly rough) that needs to be further refined in a later stage. At stage 2, however, the manual system would not receive specific stimulation enabling it to generate an answer. However, at the next stage (stage 3), the feedback transfer of articulatory information to the auditory cortex, thanks to the efference copy, would provide a more accurate answer that can now be transferred to the manual system for answer (stage 3’ in **Figure 5B**). As a consequence, RTs for manual responses would be slower than for oral response, but the responses would be more accurate because, contrary to processing for oral responses, in the manual decision mode, predictions would be confirmed and tuned in the auditory cortex before the final decision would be sent to manual motor commands (pressing the appropriate key).

Of course, this explanation is probably too simple to account for all aspects of our data. The increase in RTs with noisy stimuli (Group B), classical in any categorization experiment, requires some processing expanding over time at various stages in the loop displayed in **Figure 5**. In addition, the fact that the increase is the same in the oral and manual tasks (with no interaction between group and task for RTs) suggests that expansion should basically take place at stages 1 and 2 rather than 3 (but many variants could certainly be suggested). The crucial aspect of our results is that a pure motor translation process typical of motor theories, though compatible with faster RTs in the oral mode, does not appear in line with the associated decrease in response accuracy. On the contrary, it fits well with perceptuo-motor theories of speech perception such as the one proposed by Skipper et al. (2007; see also a computational implementation of a perceptuo-motor theory in Moulin-Frier et al., 2012). Partly in line with this hypothesis, it has to be mentioned that close-shadowing as well choral speech are well-known to be a powerful fluency enhancer that is thought to correct deficits in sensorimotor integration (i.e., weak internal modeling; see Harbison et al., 1989; Kalinowski and Saltuklaroglu, 2003).

### EFFECTS OF MODALITY: AUDIO vs. AUDIOVISUAL

Effects of modality in our study are only present in the Group B with noisy stimuli. In the auditory modality, RTs are slower than in the audiovisual modality, and proportions of correct answer are lower. Taken together, this shows a clear benefit of adding the visual modality to the auditory input, which is consistent with all previous studies since Sumby and Pollack (1954) which display an audiovisual benefit to speech recognition in noise conditions. In our study, the audiovisual advantage is present only for the /apa/ syllable which is classical and in line with the higher visibility of the lips movement associated with the bilabial /p/, and the high degree of confusion between visual movements associated with /t/ or /k/ consonants, generally considered to belong to the same visemic class. These effects of modality are not displayed in Group A with clear stimuli. This is probably because in this group, RTs in the auditory modality were already too short and proportions of correct answer were too high to be improved by the visual input (floor effect).

An interesting point is that there is no significant interaction between modality and task that is to say that the decrease of RTs and the increase of proportions of correct responses from the audio to the audiovisual modality are similar for the manual and oral tasks. Once again we will attempt to interpret this lack of interaction to the model proposed by Skipper et al. (2007).

In their model, Skipper et al. (2007) propose that the auditory and visual information, after a preliminary stage of unisensory processing respectively in visual and auditory areas, would converge in the multisensory area STp in the posterior superior temporal cortex (stage 1 in **Figure 5C**). Therefore, in case of multisensory inputs, the first hypothesis would be actually multisensory rather than uniquely auditory. From this basis, here again, a phonemic hypothesis associated with articulatory goals would be generated in POp and evoke motor commands in PMv/M1 (stage 2 in **Figure 5C**), and the efferent copy would produce in STp an auditory prediction to be compared with the auditory input (stage 3 in **Figure 5C**).In our study, audiovisual interactions in stage 1 would refine sensory processing and produce quicker and more accurate phonemic hypotheses in stage 2, which is the stage where, in our interpretation, oral responses would be generated (stage 2’ in **Figure 5B**). Then, the same gain in speed and accuracy would be propagated toward stage 3 where manual responses would be generated (stage 3’ in **Figure 5B**). Therefore, there is no strong reason to expect differences in visual gain between oral and manual tasks, the gain being essentially determined as soon as stage 1 in the model.

In summary, the results of the present study suggest that oral and manual responses are generated at two different stages in the whole perceptual chain. In the framework of an “analysis-by-synthesis” approach, manual responses would be provided only at the end of the entire loop, following motor predictions then commands themselves generating a multisensory hypothesis compared to the incident multisensory stream. However, oral responses would be produced at an earlier stage where motor commands are generated, causing faster but less precise responses. The visual input would increase speed and accuracy for sufficiently visible phonemes (e.g., /p/) in case of adverse listening conditions (such as noise). Once again, it is important to stress that other interpretations or frameworks could be provided. But globally, we argue that the whole set of results of this study seems to require a perceptuo-motor theory of speech perception in which the auditory and visual streams are integrated together and with internally generated motor representations before a final decision may be available.

## Conflict of Interest Statement

The authors declare that the research was conducted in the absence of any commercial or financial relationships that could be construed as a potential conflict of interest.
